# The Correlation of Signal to Noise Ratio Value on DPOAE with Malondialdehyde Levels in Rattus Norvegicus Diabetes Model 

**DOI:** 10.22038/IJORL.2022.62639.3151

**Published:** 2022-07

**Authors:** Maya Fitrie Nadya Lubis, Tengku Siti Hajar Haryuna

**Affiliations:** 1 *Department of Otorhinolaryngology-Head and Neck Surgery, Faculty of Medicine, Universitas Sumatera Utara, Medan, Indonesia.*

## Abstract

**Introduction:**

The aim of this study was to determine the correlation of the signal-to-noise ratio (SNR) value on distortion product otoacoustic emissions (DPOAE) examination with malondialdehyde (MDA) levels in a diabetic rat model.

**Materials and Methods:**

The subjects of this study were 25 rats. The samples were divided into 5 groups (days of confirmed diabetes): group 1 (control/non-treatment); group 2 (3 days); group 3 (6 days); group 4 (9 days); and group 5 (12 days). Samples that confirmed diabetes were assessed by DPOAE examination and subjected to MDA-level examination. The data were processed using SPSS and considered significant if p <0.05.

**Results:**

The study showed a decrease in SNR values and an increase in MDA levels for the rats, which was confirmed by diabetes. The most significant result was shown by group 5, which compared to the other diabetes groups. A post hoc test showed the significant difference SNR value in each group (p<0.05); except for groups 1 and 2, the MDA levels showed significant differences for all groups. The Pearson correlation test showed a negative correlation between SNR values and MDA levels. A significant correlation between SNR values and MDA levels was found in group 5.

**Conclusions:**

The study showed a correlation of SNR values from DPOAE examination to MDA levels in diabetes rats, indicating that there has been tissue damage (cochlea), which is characterized by a decrease in the SNR value.

## Introduction

Diabetes mellitus (DM) is a metabolic disease with a high prevalence across the world (1). DM is characterized by hyperglycemia that occurs due to the impairment of absolute insulin function because of the autoimmune destruction of pancreatic beta cells (Type 1) and relative insulin function due to a combination of impaired pancreatic beta-cell function with insulin resistance (Type 2) (2,3).

One of the complications that often occurs in DM is hearing loss (3). The relationship between diabetes and hearing loss has been found in many clinical studies (4). The literature reports a 93% rate of diabetes-related hearing loss (4). Hearing loss in diabetes is associated with damage to the cochlea that includes thickening of the stria vascularis, atrophy of the stria vascularis, and loss of outer hair cells (5).

High blood glucose increases glycated hemoglobin (HbA1c) which is produced and stored in the walls of small blood vessels, together with injury to the endothelium, which causes vessel permeability, thickening of the membrane, and abnormal growth of endothelial cells, resulting in narrowing of the vessel lumen size; the nerve then becomes malnourished and necrotic, causing tissue ischemia and hypoxia, which results in damage to one or more nerves and/or cochlear hair cells (6).

Oxidative stress (ROS) also has an important role in cell injury in hyperglycemia and can lead to an increase in free radicals. Oxidative stress acts as a mediator of insulin resistance, which then causes atherosclerotic complications and contributes to many microvascular and macrovascular complications (7). Lipids are reported to be the main targets of ROS in diabetes; hydroperoxides have toxic effects on cells, either directly or through their degradation to highly toxic hydroxyl radicals, and can react with transition metals such as iron/copper to form stable aldehydes such as malondialdehyde (MDA), which can damage the membrane cell (2,7). MDA is the end product of lipid peroxidation and is an indicator of free-radical damage (8).

Alloxan is chemically known as 5, 5-dihydroxyl pyrimidine-2,4,6-trione, which is an organic compound, urea derivative, carcinogen, and cytotoxic glucose analog (9). The use of alloxan as a diabetogenic agent in experimental animals was first reported by Dunn and McLetchie (9). Alloxan causes DM by a mechanism essentially involving the partial degradation of pancreatic beta cells (9).

The outer hair cells are responsible for auditory reception in the inner ear; therefore, the OAE is considered a useful index for assessing cochlear function (10). Otoacoustic emission (OAE) is a weak acoustic signal that is derived from the outer hair cells in the cochlea and is widely used to assess the cochlear and micromechanics function of outer hair cells using a simple, objective, highly reachable examination; it has a specific frequency, is very useful for identifying sensorineural hearing loss, and the most common use is DPOAE in diabetes patients (11,12).

The purpose of this study was to find the relationship and correlation of damage to the organ of Corti (outer hair cells) in terms of the SNR value with MDA levels in the blood of a Rattus Norvegicus diabetes model.

## Materials and Methods

This research was an ex vivo laboratory experimental study. A randomized post-test control group design was used. The maintenance of experimental animals and the provision of treatment were carried out at the Faculty of Mathematics and Natural Sciences (FMIPA) and the Terpadu Laboratory, Faculty of Medicine, Universitas Sumatera Utara. We used 25 rats with the same population strain, homogeneous in sex and age, namely adult Rattus Norvegicus Wistar strain, male sex with a bodyweight of 150–250 grams. The research has received ethical approval from the Research Ethics Commission, Universitas Sumatera Utara No. 224/KEP/USU/2020.

The sample was given an intraperitoneal injection of Alloxan (Sigma, Indonesia) at a dose of 150 mg/kgBW with a 1 cc syringe, a single dose (9). After being treated, the blood glucose level was measured in 2x24 hours in hyperglycemic conditions (blood glucose level ≥ 200 mg/dl) (13,14). The blood glucose level was measured using a blood glucose meter (Autocheck, Germany). Each sample was divided into 5 groups: group 1 (DPOAE examination before treatment/control), group 2 (DPOAE examination and MDA level on the 3^rd^ day of DM), group 3 (DPOAE examination and MDA level on the 6^th^ day of DM), group 4 (DPOAE examination and MDA level on the 9^th^ day of DM), and group 5 (DPOAE examination and MDA level on the 12^th^ day of DM).

The DPOAE examination used a GSI brand tool (Grason-Stadler, Eden Prairie, Minnesota, United States). DPOAE examination was carried out in each group after being anesthetized using Ketamine-Hameln (Hameln Pharmaceuticals, Germany) at a dose of 50 mg/kg BW combined with Xylazine 7.5 mg/kg BW (Interchemie, the Netherlands) with a 28G syringe, intraperitoneally (15). The probe on the DPOAE examination is adjusted and placed in the ear canal, ensuring that the ear canal is clean. Ratings are measured on 1.5–12 kHz frequencies. An SNR value of ≥ 6 was considered a pass and < 6 was considered abnormal/refer (12,16). After DPOAE examination, the rats were terminated for the blood draw process. The examination of MDA levels (BioAssay, USA) was carried out spectrophotometrically with a modified thiobarbituric acid (TBA) test method. Blood was stored in a refrigerator at –80°C, and 100 µL of preparation was taken and put into a 1.5cc tube. A measure of 200 µL of 10% TCA was added then centrifuged at 14,000 rpm for 5 minutes. The supernatant was taken and put into a new tube, and then a water bath or heat block was prepared at a temperature of 100°C. Centrifugation was carried out on the standard tube to form a pellet so that the MDA did not stick to the lid or tube surface. The standard tube with 200 µL was separated. To the standard and sample, 200 µL of TBA reagent was added, and the contents were mixed and incubated for 60 minutes at 100°C. The tube was cooled to room temperature, vortexed, and centrifuged, and 100 µL was taken from each tube and placed on a 96-well plate. The plate was read at 535 nm.

***Data Analysis***

The collected data were analyzed using univariate, bivariate, and multivariate methods using SPSS. The normality of the data was assessed by the Shapiro–Wilk test and ANOVA test. A post hoc test was conducted to see the differences in each group. The Pearson test was then used to observe the correlation. The statistical test is considered significant if the p value <0.05.

## Results

Table 1 shows that the longer the DM, the lower the SNR value and the higher the MDA level, with the most significant group being group 5. The ANOVA test revealed that there was a significant difference in the SNR value and MDA levels in all the groups (p <0.05).

Table 2, which presents the post hoc Games–Howell test, shows a significant difference (p<0.05) in the SNR values of all the groups, except for groups 1 and 2 (p >0.05), as well as a significant difference in MDA levels in all the groups (p <0.05).

Table 3 shows a negative correlation between SNR values ​​and MDA levels in each group, but a significant correlation between SNR values ​​and MDA levels was only found in group 5 (p <0.05).

**Table 1:** The difference in the mean value of SNR and MDA levels in each group

**Variable**	**Group**	**Mean ± SD**	**p value**
SNR	Group 1	23.4 ± 3.17	,000*
MDA	Group 2 Group 3 Group 4 Group 5 Group 1 Group 2 Group 3 Group 4 Group 5	19.0±2.35 13.9±1.31 10.1±0.65 4.40±0.43 4.35±0.09 5.12±0.03 5.27 ± 0.05 5.45±0.01 6.80±0.26	,000*

* Statistically significant (p <0.05).

**Table 2:** Post hoc Games–Howell test results for SNR values ​​and MDA levels in each group

	**Group**		**N**	**p value**
SNR	Group 1	Group 2	5	.199
		Group 3	5	.007*
		Group 4 Group 5	5 5	.003* .001*
	Group 2	Group 3	5	.026*
		Group 4 Group 5	5 5	.003* .001*
MDA	Group 3 Group 4 Group 1 Group 2 Group 3 Group 4	Group 4 Group 5 Group 5 Group 2 Group 3 Group 4 Group 5 Group 3 Group 4 group 5 Group 4 Group 5 Group 5	5 5 5 5 5 5 5 5 5 5 5 5 5	.007* .000* .000* .000* .000* .000* .000* .005* .000* .001* .003* .001* .001*

* Statistically significant (p <0.05)

**Table 3:** The results of the Pearson correlation between SNR values ​​and blood MDA levels in each group

	**Group**	**r value**	**p value**
SNR	2	-.373	.536
MDA	3	-.244	.692
	4 5	-.229 -.983	.711 .003*


## Discussion

Studies have long shown the relationship between DM and hearing loss (6). The main clinical manifestations of DM are related to glucose, lipid, and protein metabolism disorders because a body that cannot produce insulin does not work properly (6). Vascular and neural changes in the cochlea possibly cause hearing loss in DM, including thickening of the capillary walls, especially in the stria vascularis, and loss of outer hair cells (6). The blood vessels that supply the cochlea have impaired circulation (6). Hearing loss in diabetes is bilateral, sensorineural, gradual, and progressive with a high frequency (3). In experimental animal models such as mice, outer hair cell loss starts at the basal and then moves to the apex (17). This study used a dose of 150 mg/kg BW of alloxan, which was injected intraperitoneally in experimental animal samples. Research by Aluwong (2016) also induced experimental animals using alloxan at 150 mg/kg BW; this shows that alloxan at this dose causes considerable damage to pancreatic beta cells, and the insulin secreted is unable to regulate blood glucose, resulting in an increase in the blood sugar concentration (18).

DPOAE is a type of stimulated OAE that has been frequently used because it has a wider frequency interval for early detection of cochlear function damage (19); therefore, we used the DPOAE assay in this study.

This study found a decrease in the SNR value at high frequencies, namely the frequency 1500–12000 Hz, especially on the 12^th^ day of DM rats (group 5); this indicates that hearing loss or cochlear hair cell damage in diabetes occurs starting from high frequencies.

Research conducted by Erkan (2018) also found a decrease in SNR values ​​at high frequencies, namely, the frequencies of 5714 and 8000 Hz, which were examined in Wistar rats induced by DM using streptozotocin (STZ) (19). The Karbulut study (2014) also found a decrease in the SNR value on the DPOAE examination with a frequency of 250–8000 Hz in the diabetic group (20).

Research by Qujeq (2004) found a relationship between blood MDA levels with the incidence of diabetes in rats. There was a significant increase in MDA levels in the group of diabetic rats compared to the control/non-diabetic rat group (21). Gwarzo (2014), examining blood MDA levels in alloxan-induced DM rats, also found an increase in blood MDA levels in the untreated group of diabetic rats compared to other groups (22).This is in line with this study, which found an increase in MDA levels in diabetic rats (all diabetes groups). Considering the increase in MDA levels, especially on the 12th day of DM rats (group 5), it can be concluded that the longer the patient is exposed to DM, the higher the increase in MDA levels in the blood. The presence of higher MDA levels indicates an increase in lipid peroxidation and oxidative stress as one of the causes of diabetes (22).

There is a negative correlation between the SNR value and the MDA level in each group, which indicates that the lower the SNR value, the higher the MDA level in the blood. This proves that an increase in MDA levels in plasma, serum, and tissue has been shown to occur in DM, which causes tissue damage. MDA is a predictor of oxidative stress in both the blood and cochlea. The higher MDA levels indicate that the damage to the tissue and blood becomes more substantial. Therefore, it is necessary to conduct further research on the use of antioxidants that can repair tissue damage (cochlea) using MDA markers in both blood and cochlear tissue so that hearing loss due to DM can be reduced.

## Conclusion

In this study, there was a correlation between SNR values and MDA levels indicated by a decrease in SNR values, indicating the presence of tissue (cochlea) damage and an increase in MDA levels because MDA is an indicator or marker of oxidative stress.
